# 

**Published:** 1883-12

**Authors:** 


### DENTAL DEPARTMENT OF THE UNIVERSITY OF CALIFORNIA.

The second annual commencement exercises of the Dental Department of the University of California held with Medical Department at the Baldwin Theatre, San Francisco, November 13th, 1883.

The literary exercises were as follows:

Address on behalf of the Medical Department, by Prof. R. B. Kane, M. D., M. R. C. S. I. Address on behalf of the Dental Department, by Prof. C. L. Goddard, A. M., D. D. S. Valedictory on behalf of the graduating class of the Dental Department, by N. E. Price. Confirming the Degrees of Doctor of Medicine, by President of University, W. T. Reid, A. M. Confirming the Degrees of Doctor of Dental Surgery, by President of University, W. T. Reid, A. M. Administering the Hippocratic Oath to the Graduates of the Medical Department, by Prof. Beverly Cole, A. M. D., M. R. C. S. Esq., Chairman of Faculty of Medicine. Address on behalf of the Alumni Association of the Dental Department, by G. W. Sichel, M. D., D. D. S.

The following candidates received the Degree of Doctor of Dental Surgery:

John Nelson Blood, Edwin Overton Cochrane,
Charles Boxton, Russell Hopkins Cool,
Maria A. Burch, Milton Francis Gabbs,
William Edmund Price.

The number of matriculates for the session was twenty-six.

The next session will begin February 1st, 1884, and continue till the last of October, 1884.

S. W. DENNIS, M. D.
DEAN OF THE FACULTY.

### THE MARYLAND AND DISTRICT OF COLUMBIA DENTAL ASSOCIATION.

At the last annual meeting of the Maryland and District of Columbia Dental Society, held in Baltimore, in November, the following officers were elected to serve for the ensuing year.

*President,* Dr. H. C. Thompson, District of Columbia; *Vice President,* Dr. A. Price, Maryland. *Recording and Corresponding Secretary,* Dr. F. F. Drew, Maryland. *Treasurer,* Dr. W. G. Foster, Maryland. *Reporting Secretary,* J. H. Lewis, District of Columbia. *Executive Committee,* Drs. H. B. Noble, H. M. Schooley, of District of Columbia, and B. Holly Smith, of Maryland.

F. F. DREW, D. D. S., *Sec'y*
198 N. HOWARD STREET,
Baltimore, Md.

THE MARYLAND STATE DENTAL ASSOCIATION.—At the annual meeting of the Maryland State Dental Association, held October 18th, 1883, the following officers were elected for the ensuing year.

*President*, Thomas J. Smithers, of Easton, Maryland. *First Vice President*, Thomas H. Davy, of Baltimore, Maryland. *Secoud Vice President*, David Genese, of Baltimore, Maryland. *Corresponding Secretary*, William A. Mills, of Baltimore, Maryland. *Recording Secretary*, B. Merril Hopkinson, of Baltimore, Maryland. *Treasurer*, A. C. McCurdy, of Towson, Maryland. *Executive Committee*, B. M. Wilkerson, J. C. Uhler, and R. A. Hungerford, of Baltimore, Maryland.

### To Readers and Correspondents.

Communications intended for publication, and exchange journals and papers should be addressed to the Editor of the American Journal of Dental Science, 259 N. Eutaw St., Baltimore, Md. All letters relating to the business of the Journal, or containing cash remittances, to be directed to Messrs. Snowden & Cowman. Articles for publication should be received by the editor at least three weeks previous to the first month on which the number in which it is designed for them to appear, is issued.

Dental Department of the University of California........................379

Maryland and District of Columbia Dental Association........................380

EDITORIAL, ETC.

Dental Students in Baltimore City........................380

Maryland State Dental Association........................381

The Proposed Bill to Regulate the Practice of Dentistry in the State of Maryland...381

Visiting Lists for 1884—

Physician's Visiting List........................382

Physician's Daily Pocket Record........................383

Medical Record, Visiting List, and Physician's Diary........................383

A New Work on Dental Medicine........................383

☞ The American Journal of Dental Science will contain fifty pages of reading matter in each number, making a volume of about

*Six Hundred* pages,

EXCLUSIVE OF ADVERTISEMENTS.

THE

### AMERICAN JOURNAL

OF

### DENTAL SCIENCE.

The JOURNAL is issued on the first of each month and contains not less than *fifty* pages of reading matter in each number, exclusive of advertisements, making a volume of *six hundred* pages per annum.

In each number will be found Original Communications, Correspondence, Selected Articles, Monthly Summary, Bibliographical Notices and Editorial Matter, and every effort will be exerted to render the JOURNAL worthy of the patronage of the Dental profession.

A generous co-operation is respectfully solicited from the members of the profession; and as the JOURNAL has now a printing office of its own, which materially lessens the cost of its publication, it will be issued at the reduced subscription price of

**$2.50 Per Annum in Advance.**

Communications are respectfully solicited on all subjects pertaining to practice of dentistry, as well as reports of cases occurring in dental practice.

### RATES OF ADVERTISING.

| | 1 MO. | 2 MO. | 3 MO. | 6 MO. | 12 MO. |
|---|---|---|---|---|---|
| 1 Page. | $15 00 | $22 50 | $30 00 | $50 00 | $100 00 |
| ½ " | 10 00 | 15 00 | 20 00 | 30 00 | 50 00 |
| ¼ " | 7 00 | 11 00 | 15 00 | 20 00 | 30 00 |
| ⅛ " | 5 00 | 8·00 | 11 00 | 15 00 | 20 00 |

All original communications designed for publication, works for review, new instruments for notice, exchanges, &c., should be directed to the editor, 259 N. Eutaw St., Baltimore, Md.

All advertisements and subscriptions should be addressed to

SNOWDEN & COWMAN,

*Publishers of the Am., Journal of Dental Science,*

86 W. FAYETTE ST., BET., CHARLES & LIBERTY STS.,

BALTIMORE, MD.

